# 

**Published:** 1875-02

**Authors:** 


One Dollar a Year. | New York, February, 1875. | Single Numbers, Ten Cents.

### HALL'S

### Medical Adviser,

FOR THE PREVENTION OF SICKNESS AND THE CURE OF DISEASE

### WITHOUT MEDICINE.

W. W. HALL, A. M., M. D., EDITOR,

*ALSO EDITOR OF HALL'S JOURNAL OF HEALTH.*

PUBLISHED AT 234 BROADWAY, NEW YORK.

AGENTS FOR THE TRADE:

AMERICAN NEWS COMPANY, 121 NASSAU STREET,

NEW YORK NEWS COMPANY, 18 BEEKMAN STREET.

